# Downregulation of SPARC Expression Decreases Cell Migration and Invasion Involving Epithelial-Mesenchymal Transition through the p-FAK/p-ERK Pathway in Esophageal Squamous Cell Carcinoma

**DOI:** 10.7150/jca.31427

**Published:** 2020-01-01

**Authors:** Fei Zhang, Yiyin Zhang, Jie Da, Zhenya Jia, Hongyang Wu, Kangsheng Gu

**Affiliations:** Department of Oncology, the First Affiliated Hospital of Anhui Medical University, Hefei, Anhui, 230022, P. R. China.

**Keywords:** SPARC, Migration, Invasion, Esophageal squamous cell carcinoma.

## Abstract

**Purpose:** Secreted protein acidic and rich in cysteine (SPARC) is an extracellular glycoprotein overexpressed in various malignancies, including esophageal squamous cell carcinoma (ESCC), and is involved in tumor development and progression. This study was initially designed to investigate the biological roles of SPARC in ESCC cell lines by silencing SPARC expression.

**Methods:** The expression of SPARC was examined in eight human ESCC cell lines. Eca109 and HKESC cell lines with high SPARC expression were selected and transiently transfected with SPARC-targeted small interfering RNAs (siRNAs) and subsequently evaluated its impact on cell proliferation, migration and invasion *in vitro*, as well as the underlying mechanism.

**Results:** Knockdown of SPARC by the specified siRNAs in Eca109 and HKESC cell lines resulted in dramatically downregulation of SPARC expression, and significantly decreased cell migration and invasion involving epithelial-mesenchymal transition (EMT) *in vitro*. Moreover, SPARC-targeted siRNA reduced the activation of phosphorylated focal adhesion kinase (p-FAK) and extracellular regulated protein kinase (p-ERK). Furthermore, downregulation of either FAK or SPARC expression with specified siRNAs inhibited the phosphorylation of ERK and inhibited cell migration and invasion. However, decreased SPARC expression showed no impact on cell proliferation, survival or apoptosis of Eca109 and HKESC cells when comparing to control transfected groups.

**Conclusions:** These results demonstrated that downregulation of SPARC could decrease cell migration and invasion involving EMT via the p-FAK/p-ERK pathway that might serve as a novel therapeutic target against ESCC.

## Introduction

Esophageal cancer (EC) has been reported to be the sixth most common cancer and fourth leading cause of cancer death in China 1. Esophageal squamous cell carcinoma (ESCC) is the predominant and most prevalent histological subtype, with most subjects presented with locally advanced/metastatic disease 2-3. Although numerous advances in the treatment of ESCC have been achieved in last decades, it still remains one of the most deadliest diseases 1. Accordingly, the poor prognosis is partially attributable to the local invasion of primary tumors, which can lead to increased recurrent or metastatic diseases 4. Although there is still little known about the underlying mechanism of ESCC invasion, one of the implicated proteins is secreted protein, acidic and rich in cysteine (SPARC/osteonectin/BM-40).

SPARC is a nonstructural and extracellular glycoprotein that paly vital roles in tissue development, remodeling, wound repair and angiogenesis 5. Besides, SPARC has also been documented to be involved in multiple malignant tumor behaviors, including cell proliferation, apoptosis and invasion 6-7. Moreover, overexpression of SPARC has been identified in various malignancies, such as breast cancer 8, prostate cancer 9 and glioblastomas 10. Whereas it was observed at lower level in other types of solid tumors, such as colorectal cancer 11, ovarian cancer 12 and pancreatic cancer 13. It is hypothesized that the different expressions and functions of SPARC might be attributed to the specified tumor type and surrounding environment of the tumor cells.

Both SPARC mRNA and protein have been observed in cancer and stromal cells of ESCC, and overexpression of SPARC was significantly associated with metastasis and poor prognosis 14-16. Yamashita K, et al. found that SPARC was immunolocalized intensely in carcinoma and stromal cells, whereas normal esophageal mucosa and submucosa did not express SPARC, and high expression of SPARC mRNA was closely related to lymph node metastasis and unfavorable survival in 48 EC patients 14. In addition, Che YQ, et al. demonstrated that SPARC protein was located in the cytoplasm and nuclei of ESCC cells, and it was up-regulated in ESCC and significantly associated with distant metastasis of ESCC 15. Moreover, Chen YL and his colleagues also found that the expression level of SPARC was 5.78-fold higher in cancer tissues, and high SPARC expression in ESCC parenchyma was related to lymph node metastasis and poor prognosis 16. Thus, SPARC might serve as a therapeutic target in ESCC.

However, the biological functions of SPARC in ESCC cells and the precise molecular mechanisms remain to be determined. Therefore, this study was initially designed to investigate the biological roles of SPARC in ESCC cells and explore its potential underlying mechanisms.

## Materials and Methods

### Cell lines and culture

ESCC cell lines (HKESC, Eca109, KYSE30, KYSE140, KYSE410, KYSE510, KYSE150, EC9706) used in this study were obtained from the Shanghai Cell Institute Country Cell Bank and maintained in Dulbecco's modified Eagle's medium (Gibco BRL, Grand Island, NY, USA), supplemented with 10% heat-inactivated fetal bovine serum (ThermoFisher Scientific, Carlsbad, California, USA), penicillin and streptomycin in a humidified atmosphere of 5% CO_2_ at 37℃.

### Reagents and antibodies

Antibodies against human SPARC (Invitrogen, Carlsbad, USA), E-cadherin, Vimentin, Snail, PARP, Cleaved-PARP, Caspase 3, Cleaved-caspase 3, Bcl-2, Bax, FAK, p-FAK, AKT, p-AKT, ERK, p-ERK, *β*-actin and GAPDH (Cell Signaling Technology, Beverly, MA, USA) were used for immunoblotting.

### siRNA transfection

SPARC-targeted siRNA and control siRNA were purchased from Ribobio (Guangzhou, China). Briefly, 3×10^5^ cells were seeded per well in a 6-well plate and maintained overnight. Then equimolar amounts of siRNAs were transfected using Lipofectamine 3000 (Invitrogen, Carlsbad, USA) as per the manufacturer's instructions. Cells were incubated for 48h before experiments.

### Quantified real-time reverse-transcription PCR (qRT-PCR)

Total RNA was obtained using TRIzol regent (Life Technologies, Carlsbad, California, USA) and was reversely transcribed to cDNA using a Takara kit (NHK, Japan). The mRNA expression levels were determined by qRT-PCR using a LightCycler 480 instrument (Roche Diagnostics, Basel, Switzerland) in accordance with the manufacturer's instructions under the following conditions: 95℃ for 5 min, and 50 cycles of 95℃ for 10s and 60℃ for 30s. The primers used for the qRT-PCR were as follows: SPARC, forward 5'-CCCATTGGCGAGTTTGAGAAG-3' and reverse 5'-CAAGGCCCGATGTAGTCCA-3'; GAPDH, forward 5'-CTGGGCTACACTGAGCACC-3' and reverse 5'-AAGTGGTCGTTGAGGGCAATG-3', Data were analyzed using the 2^-△△ct^ method, and GAPDH was used as normalized control.

### Western blot analysis

Briefly, proteins were extracted with radioimmunoprecipitation assay (RIPA) buffer and quantified by a BCA assay according to the manufacturer's instructions. Twenty micrograms of total protein was separated by SDS-PAGE and transferred onto a PVDF membrane. The membrane was then incubated with specified antibodies overnight at 4℃. After incubation with a peroxidase-linked secondary antibody, enhanced chemiluminescence (SuperSignal ECL, ThermoFisher Scientific, Carlsbad, USA) was utilized to visualize the protein expression levels in the membrane.

### MTS assay

To evaluate the effect of SPARC siRNA on cell viability and proliferation, we used a 3-(4,5-dimethyl-2-thiazolyl)-2,5-diphenyl-2-H-tetrazolium bromide (MTS) assay. Briefly, the cells were seeded at a density of 3×10^3^ cells per well in 96-well plates. After incubation for 72 h, 20 μl MTS was added to each well and incubated for 2h. The absorbance was recorded on the microplate reader at a 490 nm wavelength. And the assay was repeated in triplicate.

### Transwell migration and invasion assays

Transwell chambers (Corning, New York, USA) were utilized to evaluate the migration and invasion ability of cells. Briefly, for the migration assay, 1×10^5^ ESCC cells transfected with SPARC/FAK siRNAs or control were resuspended in 200 µl serum-free medium and added to the top chamber without matrix gel. Then, 500 µl serums were added to the lower compartment. After incubation for 24 h, cells remaining on the upper side were removed, and the migrated cells were fixed and stained by 0.1% crystal violet for analysis. Similarly, the invasion assay was performed as described above but with Matrigel pre-coated on the inserts and cultured for 36 h. The number of migrating or invading cells was calculated microscopically by five independent fields. The data were analyzed in triplicate assays.

### Cell apoptosis assays

Cell apoptosis were determined by Annexin-V/PI (KeyGEN, Nanjing, China) staining, followed by flow cytometer analysis (Beckman Coulter, California, USA) according to the manufacturer's instructions. The experiment was triplicately repeated.

### Statistical Analysis

Data was presented as mean expression levels (± SD). Statistical analysis was performed with student's t-test using the SPSS software version 17.0 (SPSS, Inc., Chicago, IL, USA). All comparisons were two-sided and considered with statistical significance at *P* < 0.05.

## Results

### Expression of SPARC in ESCC cell lines

**Figure 1:**
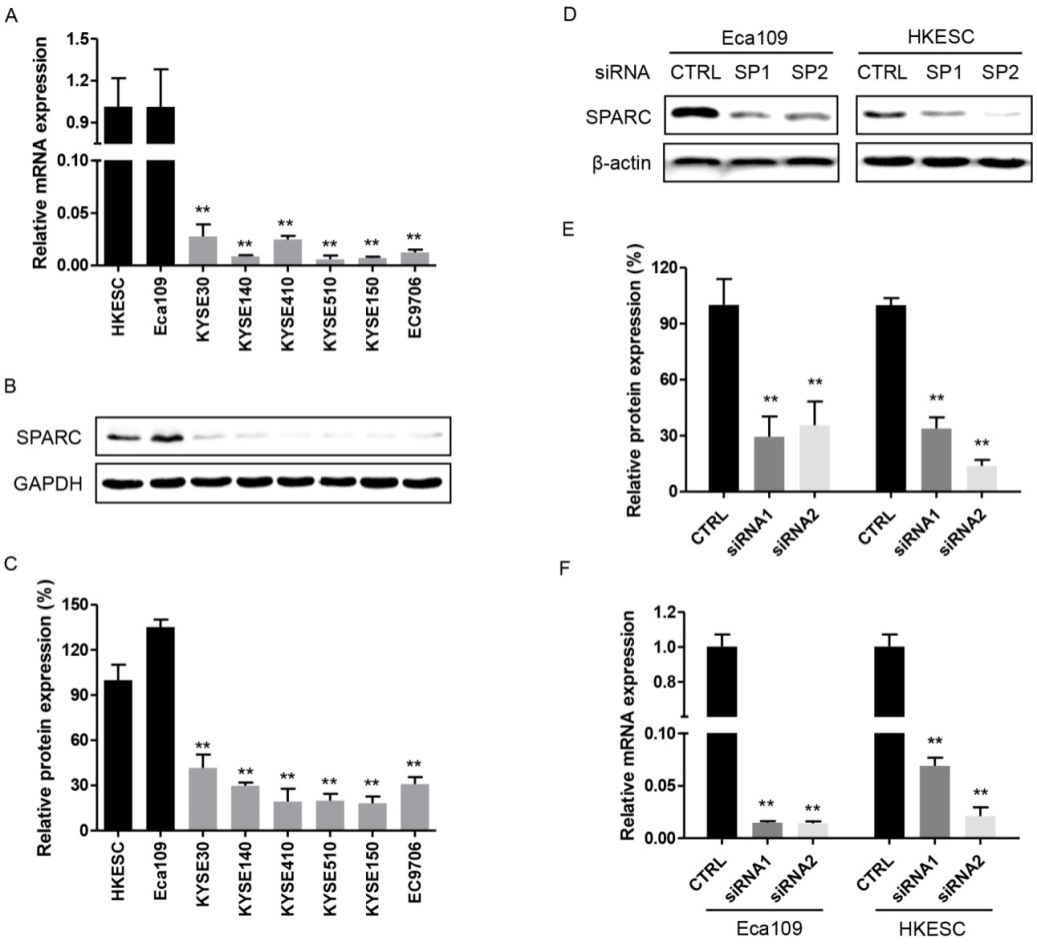
Expression of secreted protein acidic and rich in cysteine (SPARC) in esophageal squamous cell carcinoma (ESCC) cell lines, control and SPARC siRNA transfected cells. **(A) and (B)** The relative mRNA and protein expression levels of SPARC were determined using real-time RT-PCR and western blot analysis for eight ESCC cell lines. GAPDH was used as an internal control. **(C)** Quantification of western blot analysis for SPARC expression in eight ESCC cell lines. **(D)** and **(F)** The relative mRNA and protein expression levels of SPARC in Eca109 and HKESC cells transfected with equimolar quantities of siRNA of a non-targeting control (CTRL) or SPARC (SP). *β*-actin was used as loading control. **(E)** Quantification of western blot analysis for SPARC expression in Eca109 and HKESC cells transfected with equimolar quantities of siRNA of a non-targeting control (CTRL) or SPARC (SP). All experiments were performed in triplicate for each condition. All error bars represent s.d., n=3. Student's *t*-test was used for statistical analysis. ^**^P<0.01.

The endogenous expression of SPARC in eight human ESCC cell lines was initially examined. And the results showed that both SPARC mRNA and protein were overexpressed in Eca109 and HKESC cells. Whereas they were detected at lower level in the remaining six ESCC cell lines (Figure 1A-C). Therefore, both Eca109 and HKESC cell lines were selected for subsequent RNA interference studies.

### Successful inhibition of endogenous SPARC expression with specific small interfering RNAs

As shown in Figure 1D-F, the expression of SPARC was significantly inhibited with transfected SPARC siRNAs both in protein and mRNA levels. The results strongly suggested that these transfectants specific for SPARC could successfully exert a comprehensive silencing effect on SPARC expression.

### Decreased SPARC expression inhibited the migration and invasion of ESCC cells *in vitro* partially through inhibition of EMT

**Figure 2:**
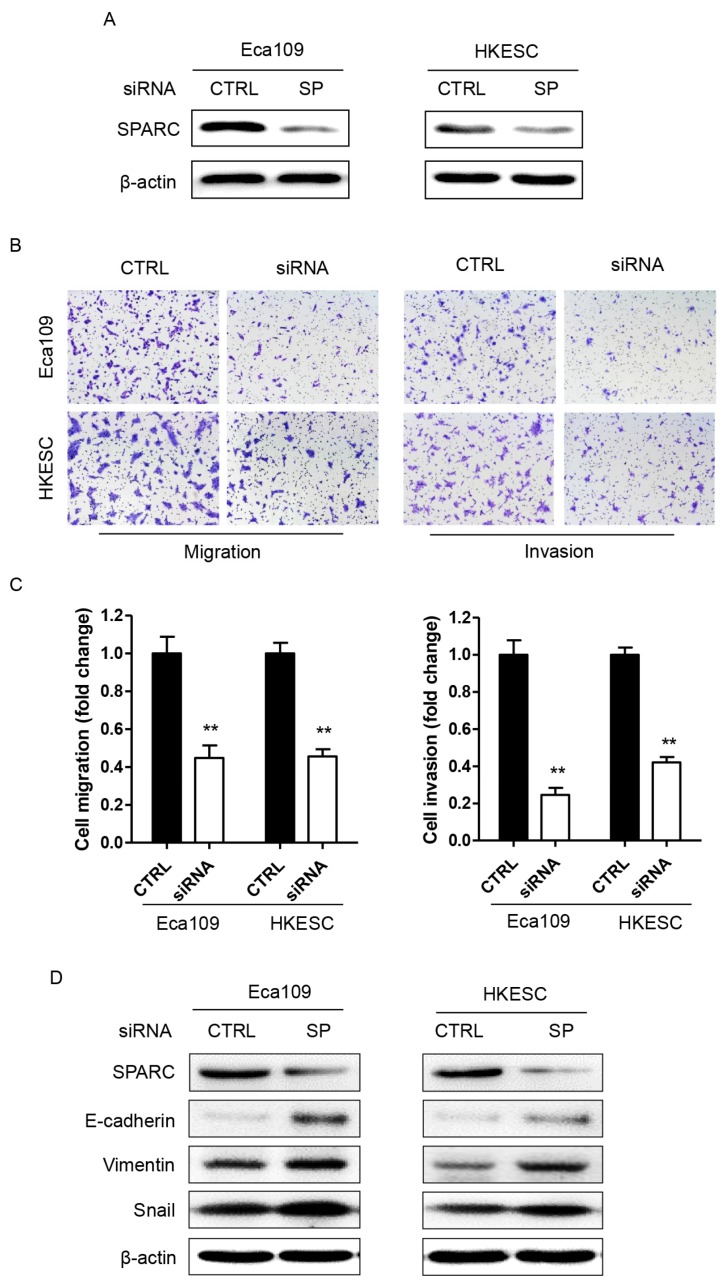
RNA interference of SPARC expression decreases ESCC cellular migration and invasion involving epithelial-mesenchymal transition (EMT). **(A)** Western blot analysis confirms successful targeting of SPARC expression in Eca109 and HKESC cells after transfection with equimolar quantities of siRNA of a non-targeting control (CTRL) or SPARC (SP). *β*-actin was used as loading control. **(B) and (C)** Representative images of migrating and invading Eca109 and HKESC cells are displayed and numbers of migrating and invading Eca109 and HKESC cells quantified. **(D)** Representative images of western blot analysis of biomarkers for EMT: E-cadherin, Vimentin and Snail. *β*-actin was used as loading control. SPARC siRNA compared with control siRNA. All error bars represent s.d., n=3. Student's *t*-test was used for statistical analysis. ^**^P<0.01.

We first evaluated the effect of downregulation of SPARC expression on the ability of tumor cell migration and invasion. As shown in Figure 2B-C, suppression of SPARC expression led to the inhibition of migration and invasion by 56% and 76% in Eca109 cells, respectively. Meanwhile, similar results were observed in HKESC cells. Moreover, as the phenotype of EMT is correlated with the metastasis of cancers, two EMT biomarkers, E-cadherin and Vimentin were detected in ESCC cells transfected with SPARC siRNAs using western blotting. And the results demonstrated that the level of Vimentin, a marker of mesenchymal cells, was significantly increased both in Eca109 and HKESC cells, whereas the expression of E-cadherin, an epithelial cell marker, was severely decreased (Figure 2D). Thus, downregulation of SPARC expression by SPARC siRNAs could decrease the migration and invasion of ESCC cells *in vitro* partially through inhibition of EMT.

### SPARC siRNA demonstrated no effect on growth or apoptosis of ESCC cells

**Figure 3:**
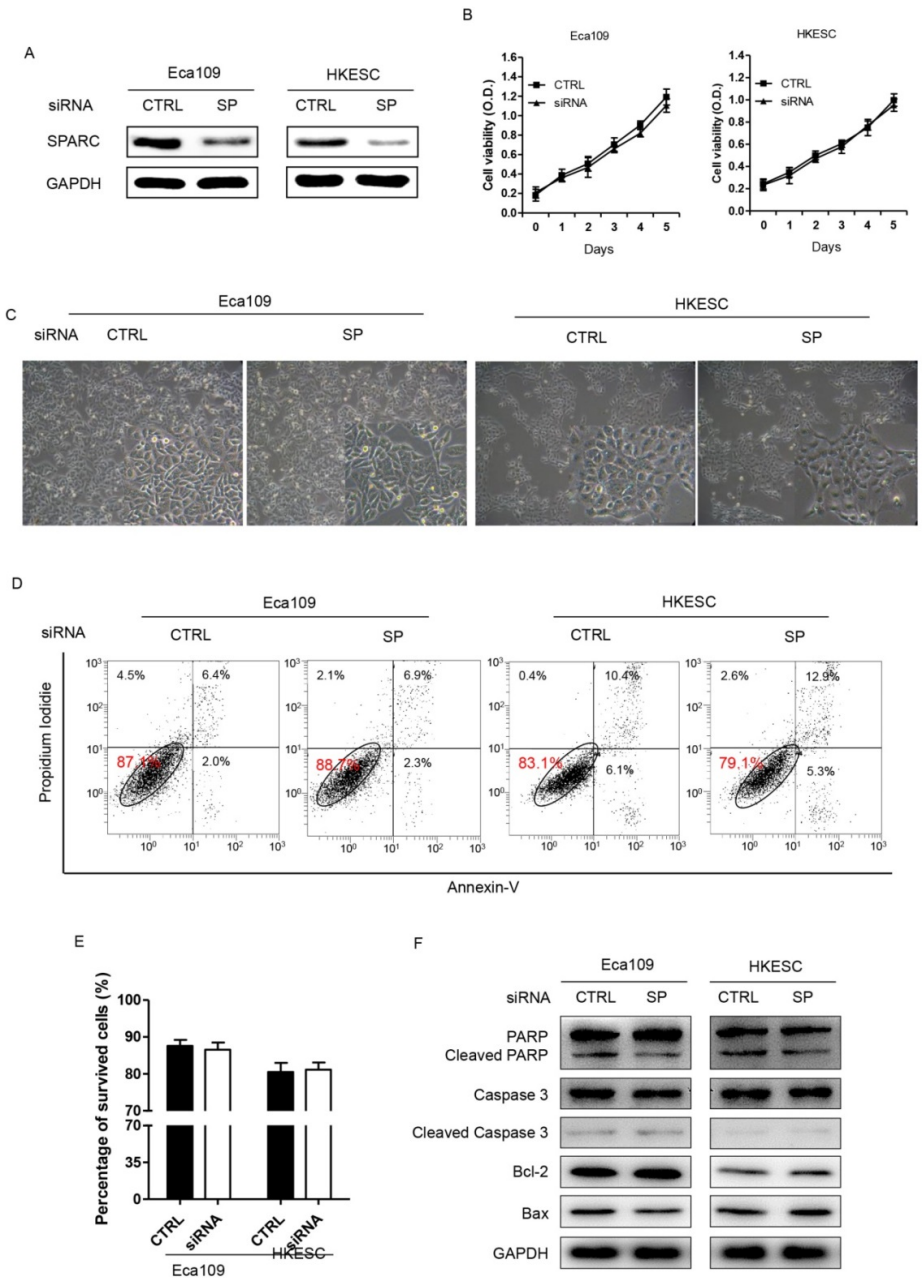
RNA interference of SPARC expression demonstrates no effect on the proliferation, survival or apoptosis of Eca109 and HKESC cells. **(A)** Western blot analysis confirms successful targeting of SPARC expression. GAPDH was used as an internal control. **(B)** Basal growth was determined after 72h in complete medium by the MTS assay. **(C)** Representative images of surviving Eca109 and HKESC cells in the survival test are displayed. **(D) and (E)** Plots of Annexin V/propidium iodide stain cell population are shown and numbers of apoptotic (Annexin V positive) cells quantified. **(F)** The expression of apoptosis proteins in Eca109 and HKESC cells after transfection with equimolar quantities of siRNA of a non-targeting control (CTRL) or SPARC (SP). GAPDH was used as an internal control. SPARC siRNA compared with control siRNA. All error bars represent s.d., n=3. Student's *t*-test was used for statistical analysis.

Eca109 and HKESC cells transfected with SPARC siRNA were then used to examine whether decreased expression of SPARC could inhibit the growth and induce the apoptosis of ESCC cells. The results suggested that both Eca109 and HKESC cells survived at equal rates relative to matched cells transfected with a non-targeting control siRNA (Figure 3A-C). In addition, the rates of apoptotic cells showed no difference between groups in both cells as measured by the Annexin-V/PI assay (Figure 3D). Furthermore, the expression of several apoptosis related proteins such as PARP, Cleaved-PARP, Caspase 3, Cleaved-Caspase 3, Bcl-2 and Bax were examined. However, compared to the control group, no significant difference in the expressions of these markers in both of the cells was observed. Thus, downregulation of SPARC expression demonstrated no effect on growth or apoptosis of ESCC cells.

### The p-FAK/p-ERK pathway was involved in SPARC-mediated cell migration and invasion

**Figure 4:**
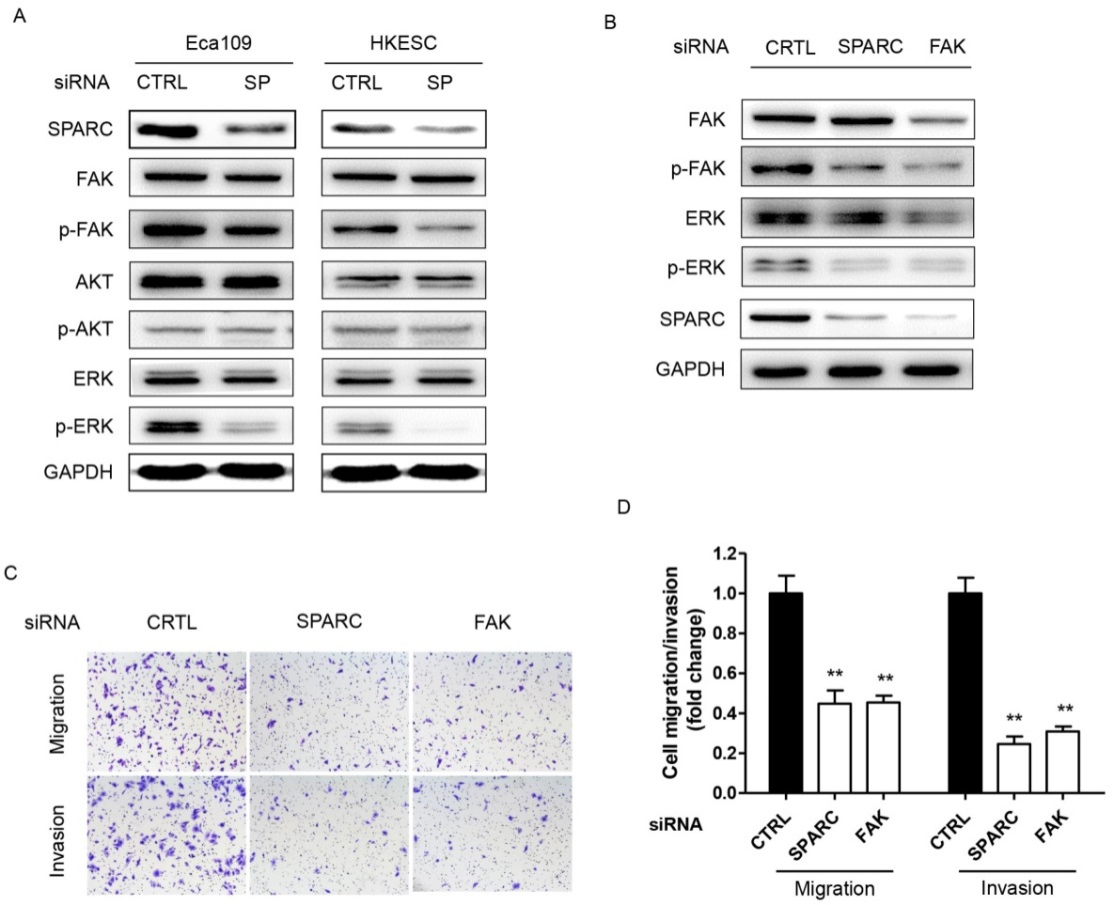
Downregulation of SPARC expression is associated with decreased phosphorylation of cellular signaling effectors, and SPARC-induced migration and invasion are FAK dependent. **(A)** Phosphorylated and total levels of FAK, AKT and ERK, as well as levels of SPARC were assessed by western blot analysis in Eca109 and HKESC cells after transfection with equimolar quantities of siRNA of a non-targeting control (CTRL) or SPARC (SP). GAPDH was used as an internal control. **(B)** Single treatment with FAK siRNA partially inhibited SPARC-induced ERK phosphorylation. GAPDH was used as an internal control. **(C) and (D)** Representative images and numbers of migrating and invading Eca109 and HKESC cells are displayed and quantified. SPARC and FAK siRNAs compared with control siRNA. All error bars represent s.d., n=3. Student's *t*-test was used for statistical analysis. ^**^P<0.01.

We next sought to determine the underlying mechanism through which SPARC siRNA could influence the migration and invasion of ESCC cells. In cells transfected with specific SPARC siRNAs, the phosphorylation of FAK (p-FAK) and ERK (p-ERK) significantly decreased, whereas no changes were detected in the levels of FAK, total AKT, phosphorylated AKT and ERK (Figure 4A). Thus, p-FAK and p-ERK are potential components of the SPARC intracellular signaling pathway. Moreover, siRNA targeting the expression of FAK also significantly inhibited the migration and invasion of ESCC cells. There were 50% and 70% decrease in the ability of SPARC-expressing Eca109 and HKESC cells to invade through the Matrigel with the transfection of FAK siRNA, respectively (Figure 4B-D). Furthermore, the phosphorylation of ERK also significantly decreased when transfected with FAK siRNA. Thus, downregulation of SPARC expression could decrease cell migration and invasion partially through the inhibition of p-FAK/p-ERK pathway in ESCC.

## Discussion

SPARC is an extracellular glycoprotein that affects tumor cell proliferation, apoptosis, angiogenesis and invasion and has been reported to be overexpressed in a broad range of human malignancies, including ESCC, upon obtainment of invasive or metastatic behaviors 5-14. Several studies have shown that the mRNA and protein levels of SPARC were higher in cancer tissues compared with the corresponding adjacent normal epithelium in ESCC, and were significantly associated with local invasion, distant metastasis and poor prognosis 14-16. However, its biological roles in ESCC cells remained largely undetermined.

To the best our knowledge, the current study demonstrated for the first time that decreased SPARC expression could significantly inhibit the migration and invasion of ESCC cells *in vitro* via suppression of epithelial-mesenchymal transition (EMT) through a novel signal transduction pathway involving SPARC, FAK, and ERK. The results were similar with previous studies. Schultz et al. and Rich et al. suggested that SPARC could increase the invasion of human glioma cell lines both *in vitro* and *in vivo* 17-18. Recently, Shi Q and his colleagues showed that downregulation of SPARC expression with siRNAs significantly decreased the invasion of glioma cells. Moreover, they found that SPARC siRNA reduced the activating phosphorylation of AKT and two cytoplasmic kinases, focal adhesion kinase (FAK) and integrin-linked kinase (ILK), suggesting that decreased SPARC-mediated AKT activation correlated with a reduction in SPARC-dependent invasion upon the suppression of FAK and/or ILK expression 19-20. Furthermore, results from Yin J, et al. revealed that siRNA-mediated knockdown of SPARC in MGC803 and HGC27 gastric cancer cells dramatically decreased their invasion 21. However, they did not evaluate the underlying mechanisms.

Unfortunately, this study failed to demonstrate the effect of SPARC expression on the growth or apoptosis of ESCC cells. On the one hand, Yin J, et al. showed that compared to the controlled groups, knockdown of SPARC could significantly inhibit the growth and increase the apoptosis of MGC803 and HGC 27 gastric cancer cells. Moreover, they suggested that the induction of apoptosis was partially attributed to mitochondrial pathway such as activation of the caspase pathway and cleavage of PARP 20. On the other hand, Shi Q, et al. demonstrated that SPARC could also promote cell survival through the activation of AKT. In addition, when treated with exogenous SPARC protein, the phosphorylation of AKT was induced in a concentration-dependent manner, and it was also increased by stable overexpression of SPARC. Furthermore, they found that suppression of SPARC expression with specific siRNAs in glioma cells decreased tumor cell survival upon the downregulation of FAK and/or ILK expression 19.

Finally, our findings have additional therapeutic implications. As both SPARC and FAK were involved in the migration and invasion of ESCC cells, tumors with high SPARC and/or FAK expression might display particularly more sensitivity to these specified inhibitors. Therefore, the expression of SPARC might help pre-selection or risk stratification of patients in clinical trials of these agents. For example, breast cancer patients with high tumor SPARC expression were seriously more sensitive to an albumin-bound paclitaxel 22, potentially because of the strong binding capacity of SPARC to albumin, which could lead to the accumulation of paclitaxel in local tumor microenvironment. In addition, the successful control of melanomas by targeting SPARC expression further suggested that knockdown of SPARC in some malignancies, such as ESCC, may be one of the effective strategies for cancer therapy 23.

In conclusion, our study demonstrated that SPARC played crucial roles in tumor progression and metastasis of ESCC and downregulation of SPARC expression could decrease cell migration and invasion via suppression of EMT through the inhibition of p-FAK/p-ERK pathway that might be a novel therapeutic target against ESCC. Thus, future clinical studies targeting the activity of SPARC are warranted in certain subjects.

## References

[1] Chen W, Zheng R, Baade PD (2016). Cancer statistics in China, 2015.

[2] Arnold M, Soerjomataram I, Ferlay J (2015). Global incidence of oesophageal cancer by histological subtype in 2012. Gut.

[3] Lin Y, Totsuka Y, He Y (2013). Epidemiology of esophageal cancer in Japan and China. J Epidemiol.

[4] Higuchi K, Koizumi W, Tanabe S (2009). Current management of esophageal squamous-cell carcinoma in Japan and other countries. Gastrointest Cancer Res.

[5] Bradshaw AD, Sage EH (2001). SPARC, a matricellular protein that functions in cellular differentiation and tissue response to injury. J Clin Invest.

[6] Sage EH (1997). Terms of attachment: SPARC and tumorigenesis. Nat Med.

[7] Nagaraju GP, Sharma D (2011). Anti-cancer role of SPARC, an inhibitor of adipogenesis. Cancer Treatment Reviews.

[8] Lien HC, Hsiao YH, Lin YS (2007). Molecular signatures of metaplastic carcinoma of the breast by large-scale transcriptional profiling: identification of genes potentially related to epithelial-mesenchymal transition. Oncogene.

[9] Wong S, Crowley D, Bronson R (2008). Analyses of the role of endogenous SPARC in mouse models of prostate and breast cancer. Clin Exp Metast.

[10] Rempel SA, Golembieski WA, Ge S (1998). SPARC, a signal of astrocytic neoplastic transformation and reactive response in human primary and xenograft gliomas. J Neuropathol Exp Neurol.

[11] Yang EN, Kang HJ, Koh KH (2007). Frequent inactivation of SPARC by promoter hypermethylation in colon cancers. Int J Cancer.

[12] Yiu GK, Chan WY, Ng SW (2001). SPARC (secreted protein acidic and rich in cysteine) induces apoptosis in ovarian cancer cells. Am J Pathol.

[13] Chen G, Tian X, Liu Z (2010). Inhibition of endogenous SPARC enhances pancreatic cancer cell growth: modulation by FGFR1-III isoform expression. Br J Cancer.

[14] Yamashita K, Upadhay S, Mimori K (2003). Clinical Significance of Secreted Protein Acidic and Rich in Cystein in Esophageal Carcinoma and its Relation to Carcinoma Progression. Cancer.

[15] Che YQ, Luo AP, Wang H (2006). The differential expression of SPARC in esophageal squamous cell carcinoma. Int J Mol Med.

[16] Chen YL, Zhang Y, Tan Y (2017). Clinical significance of SPARC in esophageal squamous cell carcinoma. Biochem Biophys Res Commun.

[17] Schultz C, Lemke N, Ge SG (2002). Secreted protein acidic and rich in cysteine promotes glioma invasion and delays tumor growth *in vivo*. Cancer Res.

[18] Rich JN, Shi Q, Hjelmeland M (2003). Bone-related genes expressed in advanced malignancies induce invasion and metastasis in a genetically defined human cancer model. J Biol Chem.

[19] Shi Q, Bao SD, Maxwell JA (2004). Secreted protein acidic, rich in cysteine (SPARC), mediates cellular survival of gliomas through AKT activation. J Biol Chem.

[20] Shi Q, Bao SD, Song L (2007). Targeting SPARC expression decreases glioma cellular survival and invasion associated with reduced activities of FAK and ILK kinases. Oncogene.

[21] Yin J, Chen GW, Liu YC (2010). Downregulation of SPARC expression decreases gastric cancer cellular invasion and survival. J Exp ClinCancer Res.

[22] Gradishar WJ (2006). Albumin-bound paclitaxel: a next-generation taxane. Expert Opin Pharmacother.

[23] Alvarez MJ, Prada F, Salvatierra E (2005). Secreted protein acidic and rich in cysteine produced by human melanoma cells modulates polymorphonuclear leukocyte recruitment and antitumor cytotoxic capacity. Cancer Res.

